# Acceptability of physical activity signposting for pre-frail older adults: a qualitative study to inform intervention development

**DOI:** 10.1186/s12877-023-04202-8

**Published:** 2023-10-03

**Authors:** Annemarie Money, Danielle Harris, Helen Hawley-Hague, Jane McDermott, Emma Vardy, Chris Todd

**Affiliations:** 1 National Institute for Health and Care Research, Applied Research Collaboration Greater Manchester, School of Health Sciences, Faculty of Biology, Medicine and Health, The University of Manchester, Manchester, M13 9PL UK; 2 Manchester Academic Health Science Centre, Manchester, M13 9PL UK; 3 Manchester Institute for Collaborative Research on Ageing, The University of Manchester, Manchester, M13 9PL UK; 4 School of Health Sciences, Faculty of Biology, Medicine and Health, The University of Manchester, Manchester, M13 9PL UK; 5 Northern Care Alliance NHS Foundation Trust, Salford, M6 8HD UK; 6 Manchester University NHS Foundation Trust, Manchester, M13 9WL UK

**Keywords:** Older adults, Frailty, Pre-frailty, Physical activity, Healthy ageing

## Abstract

Frailty is a medical condition common in older adults characterised by diminished strength and reduced physiologic function in which individuals are more vulnerable to multiple adverse health outcomes. Pre-frailty is an intermediate stage associated with some minor health outcomes. However, the main risk is progression toward moderate/severe frailty. Evidence shows physical activity interventions to be effective in slowing or modifying the progression of frailty. Researchers at the University of Manchester are developing a behaviour change intervention targeting pre-frail older adults, signposting them to group-based physical activity classes known to be effective for delaying/slowing frailty. This paper reports on the initial intervention development work with key stakeholders exploring the practicality of taking forward this intervention and identifying uncertainties to be explored in the feasibility stage. These included issues around physical activity messaging, the use of the term ‘frail’, identification/recruitment of pre-frail older adults, and the acceptability of behaviour change techniques. There was overwhelming support for a proactive approach to addressing pre-frailty issues. Given that a large proportion of older adults are estimated to be pre-frail, interventions aimed at this group have the potential to support healthy ageing, positively impacting on frailty outcomes and providing wider population health benefits.

**Supplementary Information:**

The online version contains supplementary material available at 10.1186/s12877-023-04202-8.

## Introduction

Since the early 2000s there has been much interest and activity in frailty research [1–4]. Frailty is a medical condition common in older adults with multiple causes and contributors that is characterised by diminished strength, endurance, and reduced physiologic function [5, 6]. Frail individuals experience poor recovery from minor events and are more vulnerable to multiple adverse health outcomes including falls, disability, hospitalisation, moves to care homes, dementia, poor quality of life and death [7]. Recent prevalence estimates of frailty in England [8] provide a figure of 8.1% for adults aged 50+, and prevalence of frailty increases with age (e.g., 2.8% 50–54-year-olds compared to 40.8% for 90 + year olds). Associated healthcare costs are estimated to be 5–6 times higher in frail older people [9] and the impact of frailty on health and social care is likely to increase as the number of people aged 75 + in the UK continues to grow. Frailty is not a fixed condition, but rather is understood as a continuum from robust health through to mild/pre-frailty through to moderate and then severe frailty [10, 11]. Pre-frailty is an intermediate stage associated with some minor adverse health outcomes. The main risk, however, is that pre-frail individuals may be more vulnerable to progression toward moderate or severe frailty. Estimates of prevalence of pre-frailty vary. The worldwide estimate of prevalence of pre-frailty in adults aged 65 years and older is 41% [12]. Although frailty is a long-term condition, its progression is modifiable [13, 14]. Recent evidence suggests physical activity (PA) and nutritional interventions are most effective in achieving this [15–22]. More recent evidence suggests such interventions targeted at older adults with pre-frailty have the potential to be more effective in keeping frailty status stable, or even moving individuals back to robust health [23, 24].

PA is beneficial to older adults and there is strong evidence for the impact of engagement in PA throughout the lifespan [25, 26]. In older adults, PA has a protective effect on a range of chronic conditions including coronary heart disease, obesity, type 2 diabetes, risk of falls and fractures and mental health problems [27–29]. The UK Chief Medical Officer makes very clear recommendations about the benefits of PA and the amount of activity that adults should undertake each week. However, we know that older adults are the most sedentary group in the UK with just over half (57% of men and 52% of women) of adults aged 65–74 years meeting the recommended PA guidelines for aerobic activity [30]. The Covid-19 pandemic also worsened this to the detriment of health [31]. Evidence also shows that PA rates decrease with increasing age.

Under the current contract, general practices in England are required to identify all patients aged 65 and over who may be living with moderate or severe frailty. For those with moderate / severe frailty, GPs need to undertake a series of activities including a medicine review and a falls risk assessment. Currently, there is no contractual obligation to identify mild or pre-frail patients within primary care. Given that a large proportion of older adults are estimated to be pre-frail, combined with the evidence for the benefits of PA across the lifespan, interventions (PA) aimed at this group of older adults have the potential to impact on frailty outcome / progression and wider population health benefits.

**Fig. 1:**
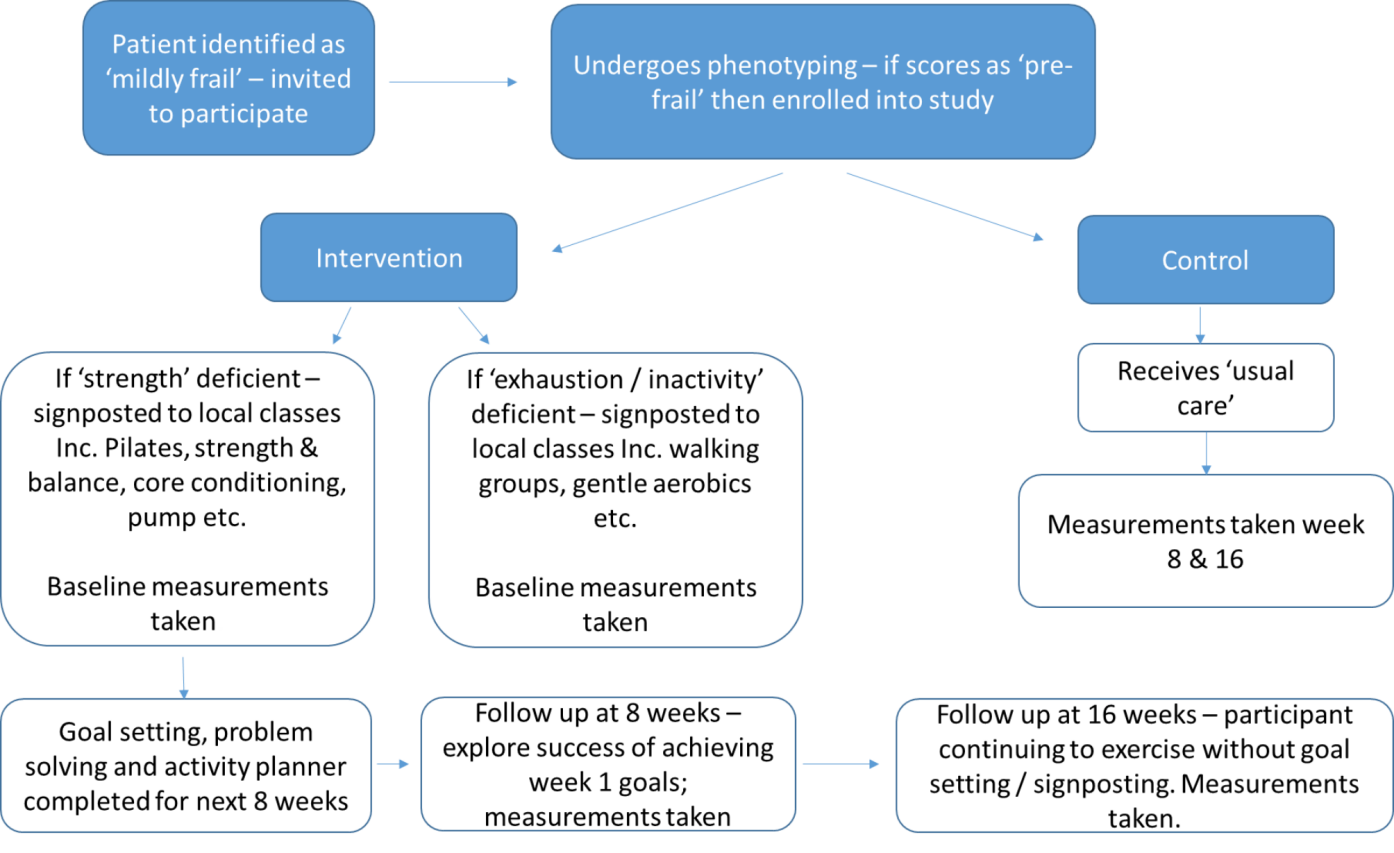
Outline of signpost to health intervention

The UK’s Medical Research Council recommends a development-evaluation-implementation model for the development and testing of complex interventions [32]. One aspect of the initial development stage is engagement with the intended intervention groups (patients / practitioners etc.) to ensure acceptability of any proposed intervention and uncover key uncertainties in the design. The ‘Signpost to Health’ project, being developed by researchers at the University of Manchester, is a signposting and behaviour change intervention that would target older adults (65 years+) identified via their primary care record as having pre-frailty. The aim of this preventative intervention would be to signpost older adults to group-based PA classes and follow them through goal setting, activity planning and monitoring of progress (see Fig. 1).

This paper reports on the formative qualitative research undertaken with two key groups; older adults and health and exercise professionals (potentially both involved in the signposting and delivery of the definitive intervention) to inform its development [33, 34]. Specifically, to explore the practicality of the proposed intervention, explore the different stakeholder groups’ views on how they think such an intervention would work in practice, what they believe may be the barriers to implementing such an intervention, and uncover any uncertainties to test during the feasibility stage.

## Methods

The study was reviewed and approved by The University of Manchester Proportionate Research Ethics Committee [Reference number 2020-9360-16503] and given Health Research Authority (HRA) approval [reference number 20/HRA/5361]. The study was conducted according to the Declaration of Helsinki. Informed consent was obtained from all participants prior to participation, and they were all assured that they could withdraw their consent at any time without consequence.

Study design: Semi-structured qualitative interviews.

Sampling, recruitment, and data collection: Purposive sampling was used to identify and recruit participants. Recruitment was via a number of third-party organisations including a Greater Manchester wide collective of leisure and community organisations, the National Institute for Health and Care Research Clinical Research Network (NIHR CRN) and the Greater Manchester Clinical Frailty Care Reference Group [35].

Data were collected via semi-structured interviews (conducted by AM) with a topic guide that was developed from key literature and from input from the project team. All interviews were virtual (by telephone or other remote means agreed with participants) at a time convenient to them. Participants gave informed consent before data collection. Data were collected between February 2021 and January 2022. All respondents were interviewed once and as soon as ‘saturation’ was reached [36], i.e., when no new insights or issues are identified during data collection, then no further interviews were undertaken.

Interviews were audio-recorded, professionally transcribed and exported to NVivo 12 Pro software for data management [37]. Applying a thematic analysis approach [38], initial themes were identified from the transcripts and indexed to develop analytical categories. Via a process of constant comparison [39], these categories were reviewed and refined by the interviewer and second researcher (DH), and any ambiguities in the coding framework were reconciled by thorough discussion with the research team. All interviews were then fully coded using NVivo 12 for qualitative analysis.

## Results

**Fig. 2:**
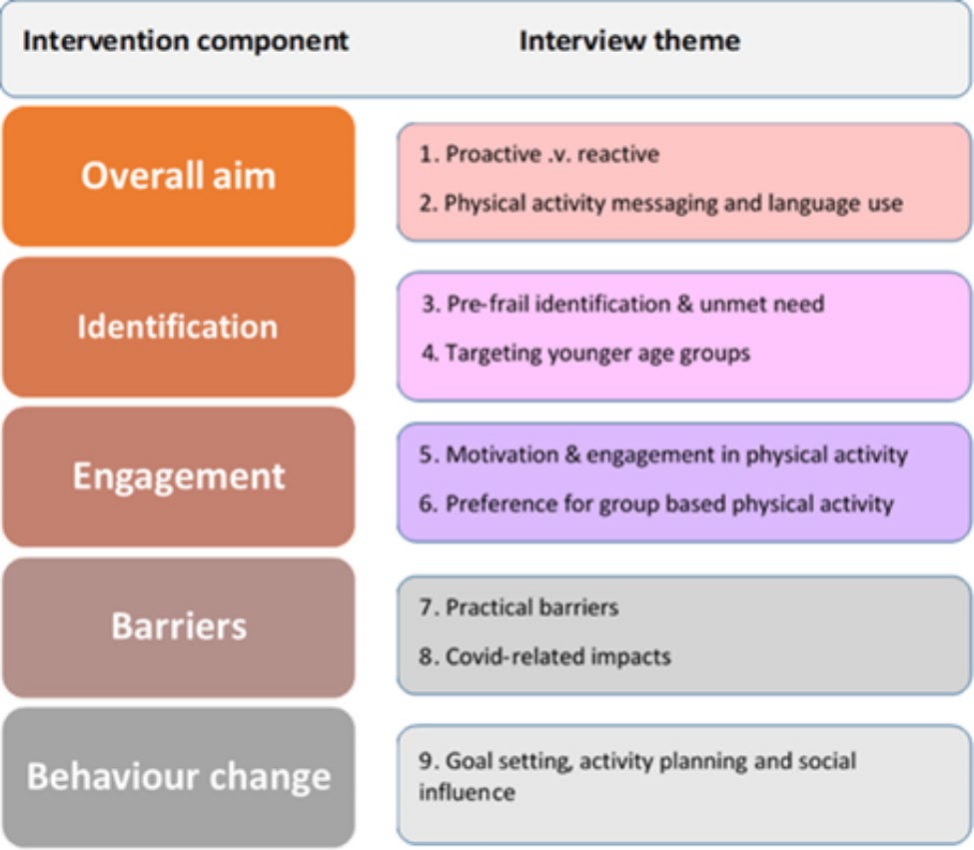
Mapping of interview themes to intervention component

In total, 43 interviews were conducted with two groups of respondents: adults over the age of 65 years (n = 22) and health and exercise professionals (n = 21 including Physical Activity Referral Officers (PARs) (n = 11), General Practitioners (GPs) (n = 5), falls prevention leads (n = 4) and a senior physiotherapist (n = 1)). Nearly three quarters of the older adult sample (16/22) were female, and the mean age (all respondents) was 74 years (range = 66 to 87 years). All were enrolled in a local PA programme either because of the rehabilitation pathway put in place after suffering from a period of ill-health, or on discharge from hospital after treatment (e.g. fall / stroke / heart attack). The interviews were structured around the broad components of the intervention design relating to (1) the overall aim of the intervention, (2) identification of pre-frail older adults, (3) engagement with PA, (4) understanding potential barriers and facilitators, (5) aspects of behaviour change. The emerging themes from the analysis have been mapped to these broad areas (see Fig. 2) and are discussed in detail below.

### Proactive .v. reactive

The overall aim of the signpost to health study is to target those adults who have yet to progress to moderate or even severe frailty, not yet had a fall or a fracture, or experienced any other health implication associated with the onset of frailty. Initial discussions around the aim of the intervention were extremely positive from all respondents involved, and the intervention was viewed as a proactive and much needed approach:

“*I think it’s a really good idea to target people before they become too frail because I think one of the major issues we’ve seen when we do see older adults… they are too frail and there’s that point where any development of condition or any regression of how they are is very hard to stop because they have gone past that point. So if we’re getting people before that point, I think it would be really good*” (PARs, 2).

“*So it’s almost a refreshing change when you see, oh, somebody’s not frail or, you know, they’re mild, so maybe we can do some work there*” (GP, 39).

“*I think it’s a brilliant idea because once you’re frail, it’s too late, in a way. It isn’t really, but just think, if you’d done it six months earlier*” (Older adult, 10).

### Physical activity messaging and language use

While the overall purpose of the intervention was welcomed by all respondents, there were a number of broad concerns relayed regarding the intervention design. These concerns relate to the issue of PA messaging and how any study materials needed careful management of both this message, and the language used to describe the intervention. In terms of PA messaging, some GPs and PARs described how this was an ongoing difficulty in managing certain conditions in their patients. There is sometimes difficulty in getting patients to understand the benefits of certain types of exercise on the management of that condition; how it could help rather than hinder;

“*And, you know, I’ve had difficulty selling that to them, and just saying, actually, no, you’ve got arthritis, but I don’t want you to do less, I want you to do more, and then, kind of, getting confused looks, sometimes*” (GP, 39).

Another difficulty is around PA messaging in terms of patients believing that the exercise they undertake currently is sufficient to help with their conditions:

“ *…just because you walk out of the house and go around the block, that that’s not enough, really …A lot of people seem to, they go, I do exercise, and then I ask them what they do and just say…well, that’s not enough really. But they seem to think…but they’re a lot more aware of healthy eating, what they should and they shouldn’t be eating, but when it comes to exercise, they’re at a loss really*” (GP, 39).

There were also discussions around language use; specifically in relation to talking about exercise versus PA, and also how the use of the terms ‘pre-frail’ and ‘frail’ were potentially problematic when describing participants. As a clinical term frailty is well-established, however outside clinical practice, negative connotations exist around the use of the term in relation to older adults;

*“I don’t mention it. Because yeah, I think it probably would have a…it might be taken the wrong way that frail might mean end of life to some patients, oh, are you telling me that this is it now, how long have I got sort of approach. Whereas that’s the last thing you want to be saying to these patients….I don’t know, frail seems to…it’s almost like…it’s worse than saying old age I think personally*” (GP, 41).

“*The only reservation I have at the moment is a little bit about the title, because I think the people you want to target, if you call them frail they do not perceive themselves in that format…. So once upon a time it was don’t mention the F word, you know, can we put that if you tell people that they’re coming up on a risk factor of frail or pre-frail, I don’t think they’ll go for it…” (Falls prevention lead, 23)*.

“*I suppose I don’t like the word. Frail, I get this picture of, you know, doddery old people, and I don’t put myself in that category….And I didn’t want to be perceived in the way that they were perceiving me…” (Older adult, 15)*.

### Identifying pre-frail participants; using the electronic Frailty Index (eFI) and creating unmet need

Health and exercise professionals raised concerns about how the intervention would work in terms of the identification of pre-frail participants, and how this proposed identification/recruitment pathway might in turn create unmet need for those involved, in particular, GPs. As noted, under the current contract, GPs are only required to identify patients aged 65 and over who may be living with moderate or severe frailty. This is done via the Electronic Frailty Index [40] which uses information already collected in the primary health care record to identify patients (aged 65 and over) who may be living with varying degrees of frailty. Currently, there is no contractual obligation to identify mild or pre-frail patients within primary care. When discussing with respondents how best to identify and recruit pre-frail patients, GPs expressed concerns regarding unmet need and how this would be handled:

“*It all comes down to workload, doesn’t it, in a GP practice? As you can imagine, we manage multiple chronic diseases and you don’t just have one element of a person’s care, you’re managing their COPD, their renal disease, their blood pressure monitoring, and sometimes I think it’s an added workload….”* (GP, 42).

“*The pre-frail is a tricky one, ‘cause even the Electronic Frailty Index actually only identifies people that are actually already frail, so to my knowledge, there isn’t a robust system to identify pre-frail patients….We do use the frailty index, but not a lot of intervention is done for the ones that are under the mild frailty, it’s often the moderate to severe frailty, that have had lots of interventions” (GP, 42).*

While acknowledging the difficulties, some GPs offered opinions about how this could be navigated within general practice, for example, acquiring the help of the practice managers, practice nurses, care navigators or social prescribing teams;

“*You don’t need to ask the GP, you need to ask the practice manager or somebody who’s doing the admin side of it. So, that’ll be easy because you’re not taking any clinician time. I think if you’re just asking for a data set then it shouldn’t be that much of an ask, you just have to get somebody who’s cooperative. It’s probably going to take an hour of their time to sort out to do a search on EMIS or TPP (electronic health record systems used within GP Practices)” (GP, 40)*.

“*And one of the great assets, I suppose, about this team, is we are very much into social prescribing and to getting people connected to community activities, exercise groups, whatever. So, we have Care Navigators, I don’t know if you know them, and they’re a brilliant resource, and I think they would fit in perfectly to something like this…” (GP, 42).*

While many of the older adults generally felt that an invitation to participate in such an intervention that came via their GP was the best route, some of the health and exercise professionals were less convinced this might be the best pathway due to a lack of capacity, and that alternative recruitment pathways outside of primary care or GP led should be explored. Some examples offered were physiotherapists and practice nurses:

“*So I think, and to be honest, we tend to get a lot more out of the physios, than we do the GPs. I don’t mean that to sound as bad as it is, but we all know GPs are busy, and they are a GP, they’re not a specialist in this area…and it’s, you know, not sort of discriminating against them, or saying anything bad, but they are, they’re very, very limited with time. And they don’t have that specialisation with certain things” (PARs, 1).*

“*I mean GPs are obviously a very strong motivator for people. I think sometimes it comes better through the practice nurse, or nurse practitioners, or somebody that has had the chance to have a longer conversation; I think those sort of appointments help…” (PARs, 5).*

### Targeting younger age groups

Related to the issue of how to best identify pre-frail participants was a discussion around whether our proposed inclusion criteria of ‘pre-frail adults over the age of 65 years’ was the best approach. Many of the health and exercise professionals (and some older adults) thought that such an intervention would have wider impact if targeted at younger adults, aged 50 years and over.

“*I wonder whether it could’ve been you know, maybe five or even ten years younger…I don’t know, just I tend to see, even say for people who are mid-50s or 60 who might develop even the smallest amount of muscle degeneration and things like that, therefore it does start to affect things like strength, balance and various other conditions” (PARs, 8)*.

“*So, I think, I’d like to see it from 40s but if we can get to 50, that’s a very good half-way measure. So, I would like to see a Falls Prevention Service actually being for prevention not a falls responsive reactive service….So I don’t think we should be waiting ‘til people have started to fall to intervene, and the markers are there. Oh, I struggle to get up the stairs, you know, I’m struggling to get off the sofa, the markers are there, you know, and that’s what I can identify, you know. So why wait from 50 to 65 for that 15 years of uneducated deconditioning to happen?” (Physiotherapist, 29).*

### Motivation and engagement in physical activity

In thinking about the intervention development, it was important that we spoke to older adults about issues around PA engagement and motivation to keep active in later life. For many, they had been referred to a local PA programme as a result of the rehab pathway put in place after suffering from a period of ill-health or on discharge from hospital after treatment (e.g. fall / stroke / heart attack). A number of key motivating factors emerged; one was around a desire to regain the quality of life they had prior to their illness and to be able to recover well, and maintain this going forward into later life.

“*Being alive, I think. No, like I say, I think the fact I’ve had two close shaves with death that, you know, you’re sort of, thankful for every day when you get up, you know and, even just doing the housework or anything like that, you know”. (Older adult, 16)*

Others spoke about the perceived benefit of PA with respect to maintaining their independence, so being able to undertake activities of daily living, being able to socialise, and being able to maintain their current lifestyle activities:

“*So it’s really wanting to…and it’s just basic things like being able to…having the strength to get out of the bath, you’ve got to have enough arm strength to get out. The thought of…anyway so that’s the motivation”. (Older adult, 27)*

“*Well actually, in the back of my mind, I’m always frightened of being stuck in the house forever waiting to die. I’ve always been a person who goes out. I like to go out. I like to go out for a coffee. I like to go to the local wine bar. I just like to go out, and I like to meet people”. (Older adult, 10)*

Fear was also perceived as a potential motivator; for some older adults this was spoken about as a fear of deterioration and how engagement in PA could help to prevent this as they aged:

“*Well what motivates me is I’m frightened of just seizing up completely and living in my armchair. So, I know perfectly well that if I don’t use it I’ll lose it, is the classic phrase. So, its fear really that drives me on to do it and I know that it does keep me moving really…” (Older adult, 37)*.

### Preference for group based physical activity classes

There was a consensus from the older adults and the PARs staff that having the classes as a group activity was key in terms of motivating them to take part in PA. Older adults largely felt if left to their own devices at home, most wouldn’t engage. In addition, if the classes included some kind of social element (e.g. time for a coffee / chat at the end), then this was welcomed by many:

“*Give them a cuppa and a biscuit, and they’ll be there….A lot of it is social, for the older people, to be honest, it is massive. Yeah…they love the tea and biscuits after a session” (PARs 1).*

*“…and another thing I think as well, I think it should be perhaps made…now, how can I put this…to let people know that the actual companionship of meeting other people at the classes, is another good thing because I’ve made a lot of friends at these classes, you know, that I’ve gone to….not making the exercise thing…well, not say not too important, but the social side just as, is important as the exercises as well, yeah, definitely” (Older adult, 16)*.

*“We’ve found, and I don’t know if you can include this because it’s not technically physical activity, so we include a cup of tea at the end of a session, they’re coming back every week. Every week. Without fail. For a lot of these people, the social side of it is more important than the exercise. If you can get them on the social side, you’ve hooked them, and they will stay with you forever, and especially the over-65s…” (PARs, 22)*.

Aligned to the social aspects of group-based activity, many older adults were keen to point out that this would only be successful if the classes offered via signposting were “pitched” correctly, e.g., making sure that the group consisted of other adults who were similar in age and ability, and ensuring the instructor was suitable for the group:

“*So, like I say, building a rapport with them, trying to understand that there’s quite often other underlying things going on as well. So it’s pitching the sessions at the right level for them. I think the social side always comes in, so not only them building their trust with the instructor but building trust with the wider group, so an inclusive group, so they want to come. But I think whatever it is, it’s about trying to pitch a session for what that individual wants and what they want to achieve and keeping it interesting as well” (PARs 17)*.

*“I suppose if there was sort of mixed ability group or if everyone else was very good and I was not, I’d find that rather humiliating and might try and find another class, but… I suppose if it was a very authoritarian approach, you know like sort of military gym instructors are portrayed, I thought, well, you know, I’m in my 70s, I can do without this” (Older adult, 25).*

### Practical barriers

For health and exercise professionals, the main barriers to the intervention related to issues around identification and recruitment, capacity and unmet need. With regard to older adults, many spoke around issues well established in the literature, for example, around practical concerns with location of classes, reliable transport to and from classes, the timing of classes (e.g., early morning or evening) and cost implications;

*“Well, I mean, I suppose finance might be, it would depend how big the cost was. I think probably a more important, bigger one would be travel, how difficult it was, and expensive, to get there” (Older adult, 25).*

*“The very, very early ones (exercise classes) would be…So, yeah, time would be difficult, and I suppose if it’s going to be for older people who are not…they’re not going to be able to rush, I don’t feel as though I can rush anymore. Then, no, yeah, and so I think travel for a lot of older adults is difficult, if they’ve not got cars or they’re having to access public transport, that makes it a barrier straightaway, really” (PARs, 2).*

*“…Venue is a big thing. If our patients have to travel, even just more than a mile, it’s an issue for them. That’s again, something you might have to consider, accessibility to these places, is it practical for people with mobility problems, if they’ve got walking aids, or a wheelchair or scooters? A lot of patients will be coming to my door with a scooter.” (GP, 42).*

### COVID-19 related impacts

A large proportion of the PARs and older adult interviews took place during the COVID-19 pandemic in the UK, and this had an impact on discussions around potential barriers to this intervention. There was an awareness from some of the respondents that the experience of being asked to shield and stay at home during the pandemic may well lead to a potential wariness for some in getting back on public transport and into activities involving large groups of people;

*“Yes, absolutely. I think people I’ve spoken to as well, with the impacts of if they’re shielding and they’ve got lots of health conditions, they’ve been shielding as well, and there’s that kind of anxiety around even going outside as well, so that reduces activity through not attending classes but just avoidance of everyday activity as well, like not getting outside for walks and things like that. So yes, it’s going to be a massive issue, I think, as well” (PARs, 2)*.

*“For some of them I think it’s probably they’ve now got to a point, some probably won’t want to go on public transport, I know that will be a massive issue” (PARs, 22).*

*“I was just thinking with COVID and that, I mean, some people may still be a bit reluctant, even though things are now opening up, to come forward because of that….they just don’t, you know, have the confidence to go out” (GP, 39)*.

In addition, some respondents had concerns about the impact of lockdowns on the physical functioning of some older adults and how any intervention might have to take into account aspects of deconditioning on this group of people;

*“So definitely everybody’s health conditions have got worse and probably quicker than we would have expected…and it’s just, I think the team have noticed in the functional assessments that everybody’s scores had come down through that, it’s definitely deterioration” (PARs, 5)*.

*“It’s going to be a challenge, yeah, in terms of starting from scratch with people maybe and looking at the deconditioning and starting at lower levels than maybe with previous…. Or maybe just having greater referral numbers of more people that have been deconditioned, so definitely. It’s going to be a challenge….” (PARs, 17)*.

*“So the change of circumstances, especially over COVID, with someone who’s not been as active, not gone shopping, not done general housework as well, as much as what they did before, it can have a massive impact in obviously muscle strength and how they feel”. (Falls prevention lead, 26)*

### Goal setting, activity planning and social influence

The final component of the intervention design that we spoke to respondents about during the interviews related to the behaviour change aspects of the intervention; specifically, goal setting and activity planning. Overall, older adults were positive about these aspects of the intervention. In particular, many older adults welcomed activity planning and noted a preference to structure their activity on a weekly basis:

*“No, I need one of those where, you know, it’s half past ten every Tuesday because it makes me at half past ten every Tuesday go. Where if it’s casual, you’d think, oh well, I’ll do it next week” (Older adult, 10)*.

*“Yes. Definitely, if I’ve got set days to do things, that’s brilliant for me, yes” (older adult, 16)*.

*“When the classes are back on I book everything in advance, yeah. It’s only something wrong with me or not feeling great that morning that I would cancel it. But that’s what I do, I book them in advance and think, yeah, let’s just go” (older adult, 30)*.

There was a mixed response to some of the other aspects of behaviour change, for example, goal setting;

*“…Goals are always difficult. I remember when we were at the pain group they asked us to think of goals and all of us said, oh God, I don’t know, you know, because actually if you’ve been put forward for a project you haven’t got any goals, you’re just cooperating with the GP and the researcher really. So, I mean I know it’s very common to do that, isn’t it, and…But it’s not easy to think up goals and usually you’re thinking them up for the benefit for the person who’s asking you really” (older adult, 36).*

However older adults were generally keen to be able to monitor / measure any progress made;

*“It’s an encouragement because if you think…sometimes you might think you’re not improving but you are because it can be a slow process. I have to stop myself sometimes and think, yeah but a month ago you couldn’t do this, you couldn’t do that. Yeah, I think it’s a kind of feather in your cap as well, to know you’ve improved, it’s a bit of satisfaction” (Older adult, 33)*.

*“Yes. ‘Cause I think that is one big thing that does give anybody that starts anything like that, the incentive to carry on….We all like to see a result, don’t we? So, yeah, yeah” (older adult, 7)*.

Discussions with PARs staff (who have experience of employing behaviour change techniques with older adults undertaking PA), highlighted that with older adults, often the process of working out the right approach took time at the start of a programme. It was important to take an individualised approach to behaviour change, and this was often front loaded and time consuming, but an important aspect of any interaction with older adults to work out which tools (diaries, goals, weekly prompts to attend etc.) might work best for different adults;

*“…We’ll sit down, and we’ll work out goals with people, you know, one to one, and what not. And we give out, obviously we do not at the moment, but we would give out little exercise diaries, so they can tick off how many sessions they’ve been to, so they’ve got that visual aid to help motivate them. So, that helps, but anything where you’re sitting down and they feel like they’re in control, or it’s their decision, would really help, I think, too” (PARs, 6)*.

*“Yeah, so it’s quite difficult….And we’ve realised we have to frontload all our support into the first 12 weeks. And it takes us on average 79 per cent of 12 weeks across all our people with a high frailty index to get them to engage and realise, ooh, this is actually a good idea…” (PARs, 4)*.

PARs staff also pointed out how social aspects of group based PA were important factors in successfully changing behaviour and promoting adherence for older adults;

“*Well, I like it, I like it. But there’s so many. It’s so individual. Some people, I might ask to keep a training diary, some people I might just always have a chat with them before or after the class, some people I might send a text message to in a week, just to check how they are or what they’ve been up to that week, or a phone call” (PARs, 3).*

## Discussion

This in-depth qualitative intervention development work was undertaken with key stakeholders to assess the practicality of putting in place a signposting and behaviour change intervention aimed at pre-frail older adults. In summary, the aim of developing the ‘Signpost to Health’ intervention with a focus on pre-frail, rather than frail older adults, was welcomed as a much-needed proactive approach to addressing issues around frailty and creating good PA habits for later life.

Important adjustments to the design of the intervention to take into account issues around language use and ensuring the messaging around PA is delivered correctly, were identified by the respondents as key concerns and will be incorporated into the final study materials. As a medical term, frailty is generally accepted by health care professionals as a clinical, deficit-based term used to refer to a long-term condition associated with ageing and characterised by an individuals’ declining resources. However, beyond medical practice, this term carries negative connotations for many older adults [41–43]. Indeed, for some older adults, being described as frail equates to a loss of independence and ultimately end of life, and this has been shown to have the potential to deter older adults (who may be becoming frail) from accessing the healthcare and support services they need [44, 45]. The intervention development work reported here highlights the need to address the use of the term ‘frail’/’frailty’ in any study materials and any conversations with potential older adult participants to ensure the focus is positive, relates to fostering independence and resilience and being able to alter or modify the trajectory/outcome rather than a label to be resisted by older adults [46]. Essentially the approach here suggests that “gain framing” [47] to emphasise the positive and avoid the negative may be more beneficial, as was shown nearly 20 years ago when presenting falls prevention services to older people, and often referred to as “Don’t mention the f-word” [48–50].

In addition, the suggestion that alternative identification and recruitment pathways for pre-frail older adults might be required, so as not to impact on already diminished capacity with the primary care system, and creation of unmet need for GPs, is an important consideration in taking the intervention to the next stage. Declining GP numbers in England [51] combined with the fact that people are now living longer lives (but not necessarily in good health) [52] has put immense pressure on the primary care system, further exacerbated by the COVID-19 pandemic. One recent approach to ease the pressure on GPs has been to integrate a workforce of non-GP roles, for example, care navigators and social prescribers, within primary care [53] and it is these personnel who were identified by GPs in this study as a potential avenue for identification and delivery of a signposting service.

In terms of the engagement components of the proposed intervention, there was overwhelming support from most of the respondents interviewed that signposting activity should be to group-based exercise classes. There has been much work in this area highlighting the impact of group-based classes on older adults’ motivation and adherence to PA [54–56] and the effectiveness of interventions adopting this approach [57]. Health professionals and older people interviewed for this development work recognise the value of a proactive intervention that will identify pre-frail older adults and signpost them to group PA with a view to preventing progression to frailty. Such interventions that are underpinned by behaviour change theory are also recognised as being effective by both practitioners and older people in this study [58, 59]. The evidence around goal setting and monitoring of behaviour is mixed for supporting PA and exercise for older adults. Personalised goal setting and feedback on behaviour have been found to be successful behaviour change techniques generally and with older adults [57–62], particularly when supporting a specific tailored exercise programme. However, when looking at more general PA, self-regulatory techniques such as setting behavioural goals and prompting self-monitoring of behaviour and providing feedback on performance have been found to be associated with lower levels of both self-efficacy and physical activity [63]. The goal setting process can be challenging to implement to ensure that goals are achievable and enhance self-efficacy. In more recent qualitative studies, it was found participants preferred to set smaller more achievable goals and to gradually modify them over time [64]. Monitoring of behaviour particularly linked to specific goals can help to support longer term adherence as it facilitates the recognition of improvements and strengthens self-efficacy [55, 65]. Interventions that are person centred and tailored to individual need are important when supporting older adults to maintain their own health, as instructors in this study have highlighted.

The study reported here has some limitations. Specifically, recruitment commenced as we entered the first lockdown in the UK [66] which resulted in a change from face-to-face data collection to online/remote methods. Also, and more importantly, it meant that we encountered difficulties recruiting GPs to interview about the intervention design as primary care at the time was focused on the COVID-19 pandemic. However, adapting our recruitment strategy allowed us to include a range of health and exercise professionals in the final sample, including GPs, falls prevention leads, physiotherapists and PARs staff. Similarly, given the difficult period in which the study was conducted, data collection was undertaken with a limited group of older adults. These older adults were all white Caucasian and already enrolled in a local PA programme through a rehabilitation pathway put in place after suffering from a period of ill-health or on discharge from hospital. In addition, they were able to use computers/digital devices as demonstrated by their ability to log onto a platform to undertake a virtual interview, although a telephone interview option was available for those who preferred. This means that our sample were already proactively engaged in managing their own health. Those who are digitally enabled are more likely to have more resources available to them and be more health literate [67]. Therefore, they may be more amenable to the intervention proposed than other older adults.

## Conclusion

A proactive behaviour change intervention aimed at signposting pre-frail older adults to group based PA classes was widely supported in this initial intervention development work with key stakeholders. Uncertainties around identification and recruitment pathways, language use, messaging and overall acceptability of the signposting and behaviour change components are to be taken forward in future feasibility work. Large proportions of older adults worldwide are estimated to be pre-frail [12], therefore an intervention of this kind, aimed at older adults who might be at risk of progression to moderate, or even severe frailty, has the potential to support healthy ageing, positively impacting on frailty outcomes and providing wider population health benefits.

### Electronic supplementary material

Below is the link to the electronic supplementary material.

Supplementary Material 1

## Data Availability

The datasets used and/or analysed during the current study are available from the corresponding author on reasonable request.
