# Supplementary material for: Acceptability of physical activity signposting for pre-frail older adults: a qualitative study to inform intervention development
Source: BMC Geriatr. 2023 Oct 3;23:621. doi: 10.1186/s12877-023-04202-8 (PMC10548637; doi:10.1186/s12877-023-04202-8)
Supplement: Supplementary file 1 — Supplementary Material 1 [file 12877_2023_4202_MOESM1_ESM.docx]

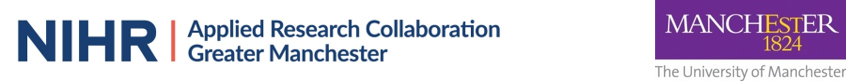


**Interview schedules for (1) older adults; (2) GP and other health professionals and (3) Physical Activity staff**

**(1) Older adults**

1.  **ONLY FOR ADULTS CURRENTLY ENGAGED IN CLASSES -** Can you tell me a little bit about your participation in this class? (Prompts: how long have you been coming, what made you come along at the start, what keeps you coming etc?)

2. **ALL RESPONDENTS -** So you’ve had time to read the summary of the project that we are developing, do you have any questions about it? What are your first thoughts about it?

3. Do you think taking part in a study like this would have encouraged you to attend this class?

**GP Identification**

4. If your GP suggested that you might benefit from a program like this that might prevent or help to reduce some aspects of frailty and build your resilience, do you think you would be willing to come along?

5. If your GP gave you the information as we have today, do you think you would find it easy to understand in terms of what would be involved in the study? Are there parts that we could explain more clearly? Or words we should use or avoid?

**Physical assessment-deficit identification**

6. How would you feel about having to undertake an assessment to find out which areas of physical activity you might need to work on? (Explain what would be involved)

7. Do you have a clear understanding of what this involves or could we explain it more clearly? (Prompt: is terminology used appropriate?)

**Signposting to exercise intervention**

8. What do you think of the type of classes that are going to be offered as part of the strengthening aspect of the intervention? (Show flashcard with exercise options)

9. Would you be interested in participating in these types of classes? (If not, then prompt for types of exercise classes they would prefer).

10. What do you think of the type of classes that are proposed to be offered as part of the increasing activity aspect of the intervention? (Show flashcard with exercise options)

11. Would you be interested in participating in these types of classes? (If not, then prompt for types of exercise classes they would prefer).

12. Would you prefer to be signposted towards exercise classes/groups, or be given sets of exercises/activities to undertake in your own time / in your own home? Reasons for preference.

**Behaviour change intervention**

13. What would you say would help to motivate you to participate in an exercise class?

14. How would you feel about planning exercise in advance? Do you think it would encourage you to attend a class? (E.g. do you think committing to 1 or 2 or 3 classes a week would help you stick to it?)

15. Do you think trying to think ahead about the barriers or things that might prevent you attending a particular exercise class might help your attendance?

16. Would you prefer to do the behaviour change aspect of the intervention as a one-to-one with a professional or as part of a group?

17. Do you have any other comments that you’d like to make about the topic we have discussed today?

_____________________________________________________________________________________________

**(2) GPs / other health professionals**

**eFI questions and current practice**

1**.** As you know from the information you have already received, the proposed intervention is planning to use the electronic frailty index to identify patients for recruitment into the study. Would you mind telling me how you currently use the eFI to identify patients who need further assessment? (Prompts: which grades do you use for deciding further assessment: mild, moderate or severe and what referral or care pathways do they progress to?)

2. We are interested in identifying those patients who are mildly frail (0.13-0.24) in your experience, is this the most practical way to identify such patients? If not, is there a better way?

3. What do you consider might be the barriers to implementing this? (Prompts: usage of eFI amongst GPs / time consuming)

4. Are you aware of any organisations that refer individuals with mild frailty to additional services? If so, what services are these?

**Providing individuals with information and referring to the intervention**

5. How would you feel referring your patients to the signposting intervention?

6. Are you comfortable using the term frail when discussing such issues with patients? (If not, ask them to explain)

7. What language would you use/avoid when referring to an individual’s level of frailty?

8. What language would you use/avoid when describing the intervention?

**Perceived uptake from those categorised with mild frailty**

9. Do you think patients in your practice, who are categorised as having mild frailty would be interested in being referred to an intervention such as this one

10. How do you think these patients would feel about undertaking physical assessment and being screened for Fried Frailty Phenotype?

11. Do you have any views on who is best placed to undertake the screening for pre frailty? Nurse, leisure centre staff, researcher?

12. Do you think there could be any language barriers to be aware of with this? If so, please elaborate on this.

**Perceived value of the intervention**

13. In what ways do you think those categorised as having mild frailty could benefit from this intervention?

14. What do you think of the proposed types of exercise classes this intervention wishes to signpost to?

**Motivational aspect of the intervention**

15. Which elements of the motivational aspect of the intervention do you think would work well?

16. What would you say are the main barriers to this intervention?

17. Do you have any ideas of how the signposting and behaviour change intervention could be improved?

18. Any other comments on anything discussed today?

_____________________________________________________________________________________________

**(3) Exercise instructors/leisure centre staff**

**Referral Plan**

1. What are your thoughts on adults classified as having ‘mild frailty’ being referred to leisure centres as part of a preventative public health intervention?

2. What do you see as the role of the GP in this? (Prompts: identification and recruitment)

3. Are there any barriers to the idea of targeting this population with an intervention signposting them to appropriate exercise groups/classes?

4. In your experience, what is the best way to effectively signpost this population to leisure services?

5. What would you normally do to screen them prior to making exercise class recommendations?

6. What current behaviour change interventions do you adopt if any? Which do you consider to be the most successful? Have you tried anything that you find does not work?

**Signposting intervention**

7. What do you think of the intervention idea to signpost individuals to a range of appropriate exercise classes based on their frailty phenotype scores?

8. Do you think there are any barriers to the language currently used in the provisional participant information sheet? Please could you elaborate on this?

9. In terms of the strengthening aspect of the intervention we are suggesting the following types of classes – LIST THEM - Do you think these classes are appropriate? If not, why? What else would you offer?

10. In terms of the increasing activity aspect of the intervention we are suggesting the following types of classes – LIST THEM - Do you think these classes/groups are appropriate? If not, why? What else would you offer?

**Motivational aspect of the intervention**

11. Do you perceive any barriers to the motivational aspect of this intervention? If so, what would they be please – could you offer any advice on how to tackle these barriers?

12. In your experience of motivating older individuals, what kind of approach seems to work best?

**Recommendations**

13. Do you have any recommendations of how this intervention may be improved?

14. Do you have any other comments you wish to make regarding the project?
